# The Effect of the COVID-19 Lockdown on the Frequency of Acute Poisoning Presentation to Adult and Pediatric Emergency Departments

**DOI:** 10.7759/cureus.33581

**Published:** 2023-01-10

**Authors:** Naif Alhussein, Meshari Alosaimi, Mohammed K Alageel, Saud M Alwatban, Rakan Aldusari, Mohammed Aldeeb, Sameerah Alsomali

**Affiliations:** 1 Medicine, King Saud Bin Abdulaziz University for Health Sciences College of Medicine, Riyadh, SAU; 2 Emergency Medicine, King Abdulaziz Medical City, Riyadh, SAU

**Keywords:** adult emergency department, pediatric emergency department (ped), lockdown, poisoning, covid-19

## Abstract

Background

The coronavirus type 2 that causes severe acute respiratory syndrome (SARS-CoV-2) was detected in December 2019 in Wuhan, China. A worldwide emergency response has been initiated because of the fast rise in the number of cases and fatalities during the early stages of the pandemic when vaccinations and efficient medical care were unavailable. Misinformation spread quickly in the early phases of the pandemic, leading to the inappropriate use of medications, chemicals, and traditional remedies for their claimed preventive or therapeutic purposes. Thus, our aim is to identify the impact of the coronavirus disease 2019 (COVID-19) lockdown on the patterns of intoxicated patients presenting to King Abdulaziz Medical City's adult and pediatric emergency departments.

Methods

A retrospective cohort study was conducted in the adult emergency department at King Abdulaziz Medical City and the pediatric emergency department at King Abdullah Specialized Children's Hospital in Riyadh, Saudi Arabia. All patients presented with poisoning as a chief complaint between March 23 to June 21 in 2019 (pre-lockdown), 2020 (the lockdown), and 2021 (post-lockdown) were included. Cases of registered poisoning complaints were reviewed and assessed with respect to patient demographics, the causative agent/substance responsible for the poisoning, management of poisoning, and particular outcomes on the approved data collection form by the research team. The data were entered and analyzed by using SPSS v26 (IBM Corp, Armonk, NY). The descriptive statistics are presented as frequency and percentage for the categorical data variables and mean and standard deviation for the numerical data. The data were further analyzed by using cross-tabulation (chi-square test), for the data that are both the predictor and the outcome was categorical. A p-value of < 0.05 was considered significant for all statistical tests.

Results

Out of 318 patients identified, 164 were adults and 154 were pediatric patients. The mean age of adult and pediatric patients was 33.3±15.5 years and 4±3.6 years, respectively. The majority of patients (115; 70.1%) were males in the adult group and females (90; 58.4%) in the pediatric. The majority of self-harm cases were accidental among both adults and pediatrics, 109 (66.5%) and 144 (93.5%), respectively. The disposition from the emergency department was discharged for 113 (68.7%) adult patients and 134 (87.0%) pediatric patients. The number of cases presented to ER with poisoning cases during the lockdown decreased. This was further tested inferentially, but no significant association was seen among study variables, i.e., p > 0.05.

Conclusion

The lockdown and pandemic had a significant impact on the rate and patterns of ED visits. The establishment and operation of Drug and Poison Information Centers (DPIC) across the Kingdom, in addition to increasing awareness through campaigns addressing drug and substance safety, is recommended.

## Introduction

The coronavirus type 2 that causes severe acute respiratory syndrome (SARS-CoV-2) was detected in December 2019 in Wuhan, China [1]. A worldwide emergency response was initiated because of the fast rise in the number of cases and fatalities during the early stages of the pandemic [2]. A pandemic was announced on March 11, 2020, by the World Health Organization in Geneva, Switzerland [3]. Misinformation spread quickly in the early phases of the pandemic, leading to the inappropriate use of substances for their claimed preventive or therapeutic purposes, despite the fact that substances are known to have detrimental and deadly consequences [4]. When it comes to medical crises like poisoning and drug overdose, knowing the rate, kind, and outcome of these cases is crucial for effective planning at both the institutional and community levels. Also, the lockdown decreased the spread of infection but it had a significant impact on people's mental health [3-5].

Globally, there where a decrease of 16% in emergency hospital visits after the declaration of the lockdown in three large hospitals in Finland, which might be attributed to the laws and restrictions implemented to decrease the spread of the disease [6]. Moreover, in Italy, compared to previous years, pediatric ER visits decreased by 73-88% [7]. In addition, poisoning was the fourth leading cause of death in rural India (where fatality rates range from 15-30%) prior to the coronavirus disease 2019 (COVID-19) pandemic. During the COVID-19 lockdown, poison centers from many countries reported an increase in calls linked to harmful exposure. Among the reported type exposures were incorrect drug usage, self-medication, and home pollutants. The majority of reported exposures, however, were to hand sanitizers, disinfectants, home cleansers, alcohol, and medications [8]. The requirement for prevention, early diagnosis, and optimal healthcare usage to decrease morbidity and death during the pandemic may be predicted by recognizing the pattern of poisonings in a specific location. World Health Organization (WHO) estimates that three million people are treated in hospitals each year after being exposed to poisonous substances; unfortunately, nearly all poisonings in third-world nations are fatal. Increasing rates of deliberate and inadvertent hazardous exposure are major contributors to high mortality in developing nations [9].

On the front line, emergency departments (EDs) play a crucial role in detecting and isolating individuals who may have COVID-19 while also providing immediate medical attention. In terms of equipment, medical personnel, bed capacity, and flexibility, hospitals, in particular the ED, must be continuously resilient and prepared to meet new crises [10].

In Saudi Arabia, however, this variance during lockdown has not been well-recorded among patients hospitalized due to hazardous exposure. Recognizing regional poisoning trends is critical for reducing morbidity and death during a pandemic by highlighting the need for prevention, early diagnosis, and proper utilization of health care. Therefore, this study aimed to identify the effect of the COVID-19 lockdown on the frequency of acute poisoning in adult and pediatric patients presenting to the ED.

## Materials and methods

This retrospective cohort study was conducted in King Abdulaziz Medical City's adult emergency department and pediatric emergency department at King Abdullah Specialized Children's Hospital in Riyadh, Saudi Arabia. Many cases of poisoning were reported at King Abdulaziz Medical City and King Abdullah Specialized Children's Hospital, which has a bed capacity of more than 1500. The emergency department contains more than 132 beds and 60 beds for adult and pediatric patients, respectively. The study aimed to assess the effect of the COVID-19 lockdown on the frequency of acute poisoning presentation in adult and pediatric patients at the emergency departments.

All patients who presented during the COVID-19 lockdown, from March 23 to June 21 in 2020 (during), with poisoning as a chief complaint were included. Moreover, patients who presented to the ED with poisoning from the matching period in the previous year, March 23 to June 21, 2019 (pre-lockdown), and from the matching period in the latter year, March 23 to June 21, 2021 (post-lockdown), were included too. The cases of registered poisoning complaints were reviewed and assessed with respect to patient demographics, the causative agent/substance responsible for the poisoning, management of poisoning, and particular outcomes on the approved data collection form by the research team.

The data were entered and analyzed by using SPSS v26 (IBM Corp., Armonk, NY). The descriptive statistic was presented as frequency and percentage for the categorical data variables and mean and standard deviation for the numerical data. The data were further analyzed by using cross-tabulation (chi-square test), for the data that are both the predictor and the outcome were categorical. A p-value of < 0.05 was considered significant for all the statistical tests. The study was approved by the Institutional Review Board (IRB) committee (NRC21R/383/09) at King Abdullah International Medical Research Center (KAIMRC), the Ministry of National Guard Health Affairs.

## Results

In total, 318 patients were identified as shown in Table 1. The mean age of adult and pediatric patients was 33.3±15.5 years and 4±3.6 years, respectively. In adults, 115 patients (70.1%) were male; however, in the pediatric age group, 90 patients were females. Regarding comorbidity, 127 patients (77.4%) in the adult group and 142 pediatric patients (92.8%) had no comorbidity.

**Table 1:** Patient demographics

		Department	
		Adult	Pediatric
Age by year, mean SD		33 +/- 3.5 years	4 +/- 3.6 years
Gender	Male	115 (70.1%)	64 (41.6%)
	Female	49 (29.9%)	90 (58.4%)
Comorbidity	Yes	37 (22.6%)	12 (7.8%)
	No	127 (77.4%)	142 (92.8%)

The majority mode in self-harm cases, as shown in Table 2, was accidental among both adult and pediatric patients, 109 (66.5%) and 144 (93.5%), respectively, followed by substance abuse and then suicidal attempts.

**Table 2:** Mode of self-harm

		Department	
		Adult	Pediatric
Mode of self-harm	Accidental	109 (66.5%)	144 (93.5%)
	Substance abuse	44 (26.8%)	8 (5.2%)
	Suicide	11 (6.7%)	2 (1.3%)

As shown in Table 3, the majority of self-harm cases were accidental among both males and females, 137 (76.5%) and 116 (83.45%), respectively. Second, substance abuse cases among males and females were 37 (20.7%) and 15 (10.8%), respectively. Moreover, males were less likely to be accidental and suicidal poisoning cases and more likely to be abuse cases. On the other hand, females were less likely to be abuse cases and more likely to be suicidal and accidental poisoning cases. This was further tested for significance association. The result showed that the mode of self-harm was significantly associated with gender (p=0.03).

**Table 3:** Mode of self-harm among males and females

		Department		P value
		Male	Female	
Mode of self-harm	Accidental	137 (76.5%)	116 (83.5%)	0.03
	Substance abuse	37 (20.7%)	15 (10.8%)	
	Suicide	5 (2.8%)	8 (5.7%)	

For disposition, as shown in Table 4, 113 adult patients (68.7%) and 134 pediatric patients (87.0%) were discharged from the ED. Regarding admission, 37 adult patients (22.6%) and 13 pediatric patients 13 (8.4%) were admitted for further observation and treatment. Furthermore, out of the 50 admitted patients, 35 adult patients (87.5) and 15 pediatric patients (100%) were discharged. Moreover, 15 patients signed LAMA (Leave Against Medical Advice), and, unfortunately, a single case of death.

**Table 4:** Disposition % Within Column LAMA: Leave Against medical advice

		Department	
		Adult	Pediatric
Disposition from ER	Discharge	113 (68%)	134 (87%)
	Admitted	37 (22.6%)	13 (8.4%)
	LAMA	10 (6.1%)	5 (3.2%)
	Referred	3 (1.8%)	2 (1.3%)
	Death	1 (0.6%)	0 (0%)
Overall hospital disposition	Discharge	35 (87.5%)	15 (100%)
	LAMA	4 (10%)	0 (0%)
	Death	1 (2.5%)	0 (0%)
	N/A	124	129

The dominant causative agent among adults was scorpion stings (59; 36%) followed by antiepileptic medications, which account for 18 (11%) in adults. On the other hand, the dominant causative agent among pediatric patients was corrosive substances (27; 17.5%) followed by acetaminophen (19; 12.3%), as shown in Table 5. As some other causative agents were dominant but not properly classified, they were added to the Other category.

**Table 5:** Causative agent % within column NSAIDs: non-steroidal anti-inflammatory drugs

	Department		
Types	Adult	pediatric	Total
Scorpion sting	59 (36%)	13 (8.4%)	72 (22.6%)
Corrosive substance	5 (3%)	27 (17.5%)	32 (10.1%)
Acetaminophen	10 (6.1%)	19 (12.3%)	29 (9.1%)
Antiepileptic medications	18 (11%)	4 (2.6%)	22 (6.9%)
NSAIDs	4 (2.4%)	11 (7.1%)	15 (4.7%)
Vitamins	2 (1.2%)	11 (7.1%)	13 (4.1%)
Asthma medications	0 (0%)	11 (7.1%)	11 (3.5%)
Alcohol	11 (6.7%)	0 (0%)	11 (3.5%)
Food poisoning	5 (3%)	4 (2.6%)	9 (2.8%)
Antihypertensive medications	3 (1.8%)	5 (3.2%)	8 (2.5%)
Amphetamine	7 (4.3%)	0 (0%)	7 (2.2%)
Detergents	1 (0.6%)	5 (3.2%)	6 (1.9%)
Carbon monoxide poisoning	6 (3.7%)	0 (0%)	6 (1.9%)
Other	33 (20.1%)	44 (28.6%)	77 (24.2%
Total	164	154	318

The number of cases presented to The ED with poisoning cases during the lockdown decreased, as shown in Table 6. This was further tested inferentially but no significant association was seen among study variables, i.e., p>0.05.

**Table 6:** Effect of the COVID-19 lockdown on pediatric and adult patients % within periods Period 1 = the first 30 days of the lockdown, Period 2 = the middle 30 days of the lockdown, Period 3 = the last 30 days of the lockdown

Pediatric	Pre	During	Post	Total	P-value
Period 1	15 (32.6%)	10 (21.7%)	21 (45.7%)	46	0.946
Period 2	15 (30.6%)	13 (26.5%)	21 (42.9%)	49	
Period 3	18 (30.5%)	12 (20.3%)	29 (49.2%)	59	
Total	48 (31.2%)	35 (22.7%)	71 (46.1%)	154	
Adult	Pre	During	Post	Total	
Period 1	21 (41.2%)	14 (27.4%)	16 (31.4%)	51	0.861
Period 2	20 (42.6%)	9 (19.1%)	18 (38.3%)	47	
Period 3	30 (45.5%)	14 (21.2%)	22 (33.3%)	66	
Total	71 (43.3%)	37 (22.6%)	56 (34.1%)	164	

## Discussion

Poisoning is known to be a major public health issue worldwide despite the variety of poisoning substances and the fact that it is influenced by culture, race, and availability. Moreover, information related to the effect of the COVID-19 lockdown on poisoning patterns is limited. This study aims to identify the impact of the COVID-19 lockdown on the patterns of intoxicated patients presenting to King Abdulaziz Medical City adult and pediatric emergency departments in order to familiarize medical practitioners with the most frequent types of acute poisoning during pandemics.

Our study showed that during the COVID-19 lockdown, there was a decrease in the number of intoxicated patients. This might be explained by the increase of laws and restrictions that were implemented during the lockdown to limit the rate of the disease spread. In contrast, Zhang et al. and Raffee et al. showed an increase in poisoning cases during the pandemic [11-12].

In this study, 90 patients (58.4%) in the pediatric ED were females compared to 64 male patients (41.6%), which is similar to Motawei et al., who showed that 58.8% of pediatric admission were females [13]. However, in the adult ED, 115 patients (70.1%) were male. Similarly, other studies conducted in India showed higher toxication rates in males than in female patients [14-15]. Furthermore, it is thought that the lockdown may have put men under stress since they are usually the primary providers in their families and were under great stress when their employment was terminated. This is in line with Behera et al., who showed 77% of adult ER visitors were males. Also, nearly 50% of the cases were unemployed, illustrating that unemployment was the primary cause of suicide during the pandemic [16].

As for toxins during the lockdown, scorpion stings were the most common in both age groups, respectively. Also, an Indian study found venomous bites present as 25% of total admissions [14]. In adults, antiepileptic drugs were the second most common observed agents. In line with similar studies that described overexposure to central nervous system (CNS)-acting drugs as the major cause of intoxications and overdoses. A number of drugs were involved in Romania, France, and Norway, with benzodiazepines being the most prevalent [17]. In the pediatric age group, the most common agent was corrosive substances, which was similar to Mintegi et al., who stated that corrosive substances and detergents were the most prevalent cause of intoxication [18]. Even though our study included a wide variety of poisonous agents that resulted in different hospital courses, the majority of patients were discharged, and one patient died secondary to the agent utilized.

Regarding the mode of self-harm, it was found that in both adult and pediatric patients, most exposures were accidental, which is similar to studies conducted in Australia, Egypt, and California [19-21]. The second most common mode of self-harm was substance abuse for both age groups, and this was compatible with another study in which there was an initial fear that the COVID-19 epidemic would lead to a large rise in alcohol and drug usage and accompanying mental health difficulties. Evidence from previous pandemics revealed that the usage can increase or decrease due to the following factors: in certain communities because of the psychological discomfort experienced or due to restricted availability and economic restrictions [22]. Suicide was the least common mode of self-harm. This study found male predominance regarding substance abuse while females exhibited more suicidal attempts. This was reported also in an Egyptian study that mentioned most male admissions were due to substance abuse. Moreover, the majority of suicidal admissions were females, which may be explained by their emotional responses to stress [13].

Our study is not without limitations. Our study has the limitation of being a retrospective analysis, so only the data that was already documented in the patient's electronic medical reports could be collected. Moreover, all patients who presented to the ED with poisoning were included even if their diagnosis required toxicology panel screening.

## Conclusions

In conclusion, the lockdown and pandemic resulted in a decrease in the rates of hazardous exposure, whether measured by the rise in calls, ED visits, or shifts in patterns. Moreover, the post-lockdown period showed an increase in the rates of poisoning cases among pediatric patients, thus selling potentially hazardous substances in containers that are childproof and having Drug and Poison Information Centers (DPIC) across the Kingdom is recommended.

## References

[1] Zhu N, Zhang D, Wang W (2020). A novel coronavirus from patients with pneumonia in China, 2019. N Engl J Med.

[2] Fisher D, Wilder-Smith A (2020). The global community needs to swiftly ramp up the response to contain COVID-19. Lancet.

[3] Nicola M, Alsafi Z, Sohrabi C (2020). The socio-economic implications of the coronavirus pandemic (COVID-19): a review. Int J Surg.

[4] Depoux A, Martin S, Karafillakis E, Preet R, Wilder-Smith A, Larson H (2020). The pandemic of social media panic travels faster than the COVID-19 outbreak. J Travel Med.

[5] Shadnia S, Darabi D, Pajoumand A, Salimi A, Abdollahi M (2007). A simplified acute physiology score in the prediction of acute organophosphate poisoning outcome in an intensive care unit. Hum Exp Toxicol.

[6] Kuitunen I, Ponkilainen VT, Launonen AP, Reito A, Hevonkorpi TP, Paloneva J, Mattila VM (2020). The effect of national lockdown due to COVID-19 on emergency department visits. Scand J Trauma Resusc Emerg Med.

[7] Lazzerini M, Barbi E, Apicella A, Marchetti F, Cardinale F, Trobia G (2020). Delayed access or provision of care in Italy resulting from fear of COVID-19. Lancet Child Adolesc Health.

[8] Chary MA, Barbuto AF, Izadmehr S, Hayes BD, Burns MM (2020). COVID-19: therapeutics and their toxicities. J Med Toxicol.

[9] Guntheti BK, Singh UP (2011). The pattern of poisoning in Khammam. J Indian Acad Forensic Med.

[10] Freund Y (2020). The challenge of emergency medicine facing the COVID-19 outbreak. Eur J Emerg Med.

[11] Zhang EW, Davis A, Finkelstein Y, Rosenfield D (2022). The effects of COVID-19 on poisonings in the paediatric emergency department. Paediatr Child Health.

[12] Raffee L, Daradkeh HM, Alawneh K, Al-Fwadleh AI, Darweesh M, Hammad NH, Almasarweh SA (2021). Impact of COVID-19 lockdown on the incidence and patterns of toxic exposures and poisoning in Jordan: a retrospective descriptive study. BMJ Open.

[13] Motawei SM, Shabka OA, Liu H (2022). Poisoning during the COVID-19 pandemic and lockdown: retrospective analysis of exposures reported to the Poison Unit of the Mansoura Emergency Hospital. Toxicol Commun.

[14] Shah SM, Asari PD, Amin AJ (2016). Clinico-epidemiological profile of patients presenting with acute poisoning. Int J Curr Res.

[15] Kumar SV, Venkateswarlu B, Sasikala M, Kumar GV (2010). A study on poisoning cases in a tertiary care hospital. J Nat Sci Biol Med.

[16] Behera A, Singla N, Sharma N, Sharma N (2022). Paradigm shift in pattern and prevalence of poisoning during COVID-19 pandemic. J Family Med Prim Care.

[17] Sorodoc V, Jaba IM, Lionte C, Mungiu OC, Sorodoc L (2011). Epidemiology of acute drug poisoning in a tertiary center from Iasi County, Romania. Hum Exp Toxicol.

[18] Mintegi S, Azkunaga B, Prego J (2019). International epidemiological differences in acute poisonings in pediatric emergency departments. Pediatr Emerg Care.

[19] Huynh A, Cairns R, Brown JA (2018). Patterns of poisoning exposure at different ages: the 2015 annual report of the Australian Poisons Information Centres. Med J Aust.

[20] Hassan BA, Siam MG (2014). Patterns of acute poisoning in childhood in Zagazig, Egypt: an epidemiological study. Int Sch Res Notices.

[21] Agran PF, Winn D, Anderson C, Trent R, Walton-Haynes L (2001). Rates of pediatric and adolescent injuries by year of age. Pediatrics.

[22] Baker TB, Piper ME, McCarthy DE, Majeskie MR, Fiore MC (2004). Addiction motivation reformulated: an affective processing model of negative reinforcement. Psychol Rev.

